# Propidium monoazide–polymerase chain reaction for detection of residual periprosthetic joint infection in two-stage revision

**DOI:** 10.1007/s11033-019-05092-z

**Published:** 2019-10-05

**Authors:** Mohamed Askar, Mariam Sajid, Yassar Nassif, Waheed Ashraf, Brigitte Scammell, Roger Bayston

**Affiliations:** 1 Department of Academic Orthopaedics, Queen’s Medical Centre, University of Nottingham, C Floor, West Block, Derby Road, Nottingham, NG7 2UH UK; 2 Trauma and Orthopaedic Department, University of Nottingham Hospitals, Nottingham, UK

**Keywords:** Propidium monoazide, Polymerase chain reaction, Periprosthetic joint infection

## Abstract

False negative culture results in periprosthetic joint infection (PJI) are not uncommon particularly when patients have received long term antibiotics. Polymerase chain reaction (PCR) has a lower specificity partly due to detection of residual DNA from dead bacteria. Propidium monoazide (PMA) prevents DNA from dead bacteria from being amplified during the PCR. This study aimed to determine the role of PMA in PCR for diagnosis of PJI. Clinical samples were tested by PCR with and without prior treatment with PMA and compared to conventional microbiological culture. The PCR assay included genus-specific primers for staphylococci and enterococci and species-specific primers for *Cutibacterium acnes.* The validated conditions of PMA treatment used in this study were 20 μM concentration and 5 and 10 min of dark incubation and photo-activation respectively. 202 periprosthetic tissues and explanted prostheses from 60 episodes in 58 patients undergoing revision arthroplasties for either PJI or non-infective causes were tested, by culture, PCR, and PMA-PCR. 14 of the 60 episodes satisfied the Musculoskeletal Infection Society (MSIS) criteria for PJI and 46 did not. Sensitivity of culture, PCR, and PMA-PCR were 50%, 71%, and 79% respectively. Specificities were 98%, 72%, and 89% respectively. All figures were calculated for episodes rather than samples. PMA-PCR enhanced both the specificity and the sensitivity of PCR. It has the potential to detect residual bacterial viability prior to reimplantation in the two-stage revision for PJI.

## Introduction

Prosthetic joint infection (PJI) is a devastating complication after joint replacement leading to great morbidity and significant burden to health care systems. It is one of the most feared modes of failure of arthroplasty because of the difficulty in diagnosis and treatment. Though the incidence of PJI according to Public Health England is currently 0.6% [1], infection accounted for 14.8% and 25.2% of revision operations after hip and knee arthroplasty respectively and was the most common cause of revision after knee replacements [2, 3]. The usual treatment for PJI is two-stage revision, where the infected joint is opened and explored, and all infected tissue and the prosthetic components are removed. At the second stage new prosthetic components are inserted but only after several weeks of antibiotic treatment to ensure as far as possible that the infecting bacteria have been eradicated. Tissue samples removed at the first stage are sent for culture, though this method has a low sensitivity. The prevalence of culture-negative PJI in the literature ranges from 7 [4] to more than 40% [5, 6].

In the last decade, PCR has been evaluated in diagnosis of PJI in an attempt to overcome the limited sensitivity of culture methods [7–9]. While PCR is a rapid and relatively sensitive technique, it can falsely indicate the presence of residual infection at stage two revisions after antibiotic treatment due to dead bacteria. DNA from dead bacteria can persist for months in clinical samples after achievement of a clinical cure [8, 10], and PCR will overestimate the number of viable bacteria as the DNA from both live and dead bacteria will be amplified indiscriminately.

Propidium monoazide (PMA) has been used to selectively limit the PCR to the viable bacteria [11–13], mainly in non-clinical settings. Propidium compounds have been known to bind to DNA and RNA for at least two decades. An added advantage is that intact bacterial cell membranes are impervious to propidium salts, allowing access only to the DNA of dead or damaged cells [14]. The presence of an azide group lets the molecule bind covalently to the DNA upon light exposure [15]. This cross-linking makes the DNA non-accessible to the polymerase elongation because of structural changes and insolubility, leading to its loss during the DNA extraction process. The remaining unbound PMA is simultaneously deactivated upon light exposure and becomes no longer capable of binding to the DNA that will be released by extraction [16].

Therefore, inclusion of PMA in PCR assay for detection of residual infection in PJI, preventing DNA from non-viable bacteria from contributing to the PCR assay, might make the assay more reliable and increase its specificity.

In this study, PCR was performed with and without PMA pre-treatment, on clinical samples from PJI and aseptic revision arthroplasties in order to investigate this.

## Materials and methods

### PMA optimisation

PMA concentration, dark incubation, and photo-activation time periods were optimised. Each one of these parameters was tested separately while the other two variables were kept constant. As a first stage, these conditions were tested on laboratory-prepared live and dead bacterial suspensions. In a second stage, to mimic a clinical situation, the optimised conditions were tested using artificially spiked human tissue homogenates for validation. Organisms used in the optimisation were isolated from PJI cases in our laboratory.

Suspensions of *Staphylococcus aureus*, being the commonest PJI pathogen, were prepared with a concentration of 10^5^ colony-forming units (cfu)/mL in phosphate buffered saline (PBS) and divided into halves. One half was killed by heating in a dry block at 95°C for 10 min while the other half was left viable. Bacterial killing was defined as a lack of growth after incubation on blood agar for 48 h.

Each of the live and dead portions was further divided into two halves; one of each was treated with PMA (Biotium, Fremont, USA) and the other half left untreated. Dark incubation was done at 37 °C in a shaking incubator for adequate mixing. The PMA-Lite™ LED Photolysis Device (Biotium) was used for the photo-activation of the PMA.

GenuElute DNA extraction kit (Sigma-Aldrich, Missouri, USA) was used for bacterial DNA extraction following the manufacturer’s protocol. PCR was run using Mx3005P QPCR (Agilent, California, USA). Each sample was run in duplicate in the same PCR assay and each test was repeated three times. For each of the live and dead samples, difference in cycle threshold Ct (ΔCt) was calculated, where: ΔCt viable is the difference between the Ct value of viable bacteria with and without PMA treatment and ΔCt dead is the difference between the Ct value of dead bacteria with and without PMA treatment. The ideal PMA treatment conditions should have the highest ΔCt dead and lowest ΔCt viable.

For PMA concentration optimisation, four different PMA concentrations were tested: 10, 20, 50 and 100 μM. After addition of the PMA, samples were incubated in the dark for 5 min and then photo-activated for 20 min.

For optimisation of dark incubation, a concentration of 20 μM was used. 5, 10, and 30 min were tested with the same photo-activation time of 20 min.

For photo-activation optimisation, a concentration of 20 μM and a five-minute dark incubation were used. 10, 20, and 30 min were tested.

### Application of optimised PMA in clinical samples

**Fig. 1:**
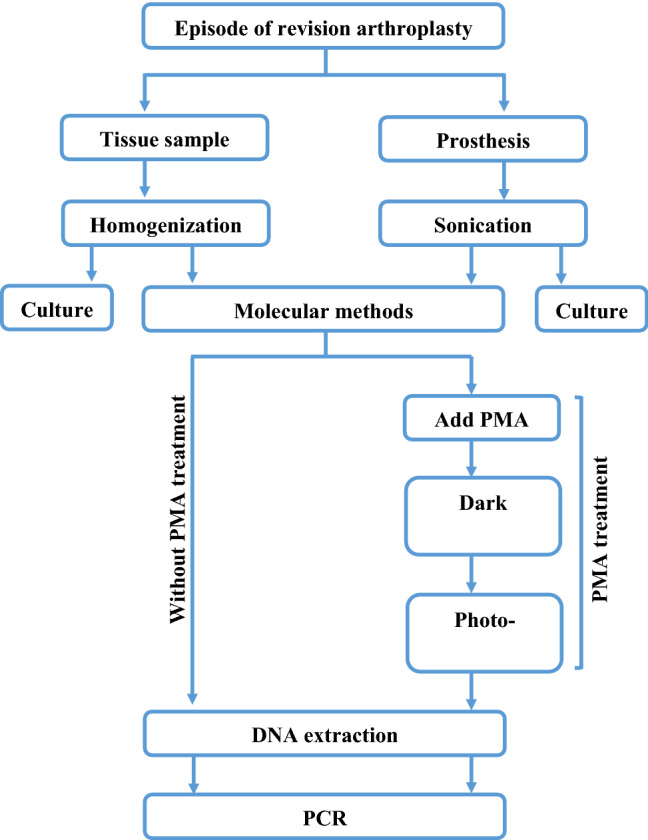
Flowchart of processing of clinical samples

Using the optimum conditions above, clinical samples were examined. 202 periprosthetic tissues and/or explanted prosthesis samples were collected from 60 episodes in 58 patients undergoing revision arthroplasties due to either PJI or non-infective causes (eg aseptic loosening or periprosthetic fracture). The Musculoskeletal Infection Society (MSIS) definition of PJI was used [17]. Figure 1 is an overview of the processing of clinical samples.

Tissues were homogenized using the Roche magNA Lyser homogenizer (Hoffman-La Roche Ltd, Basel Switzerland) at a speed of 4500 rpm for four cycles, each lasting 45 s [18]. Prostheses were sonicated in a precision sonicator set at 50 Hz (Ultrawave Ltd, Cardiff, UK) for 5 min.

Homogenates and sonicates were used for aerobic and anaerobic cultures, PCR (PCR without PMA), and PMA-PCR (viability PCR). For PMA treatment, 5 µL of 2 mM of the photo-reactive dye was added to the 500 mL sample in a dim-light room for a final concentration of 20 μM/µL. The sample was then incubated in the dark for 5 min (covered with aluminium foil), while gently shaking the tubes to ensure proper mixing. PMA-Lite™ LED Photolysis Device was used for the photo-activation for 10 min.

For DNA extraction, the GenuElute DNA extraction kit was used according to the manufacturer’s instructions.

The PCR assay included genus-specific primers for staphylococci [19] and enterococci [20] and species-specific primers for *Cutibacterium acnes* [21]. The human glyceraldehyde-3-phosphate dehydrogenase (GAPDH) housekeeping gene [22] was amplified as an internal control of DNA extraction and possible PCR inhibition (Table 1). Separate PCR assays were used for each primer pair.

**Table 1:** Sequences of primers used in the study

Bacteria	Gene	Primer sequence (5′→3′)	References
Staphylococci	*tuf* gene	Forward: CAATGCCACAAACTCG Reverse: GCTTCAGCGTAGTCTA	[19]
Enterococci	23S rRNA	Forward: AGAAATTCCAAACGAACTTG Reverse: CAGTGCTCTACCTCCATCATT	[20]
*C. acnes*	16S rDNA	Forward: GGGTTGTAAACCGCTTTCGCCT Reverse: GGCACACCCATCTCTGAGCAC	[21]
	GAPDH	Forward: TCCCTGAGCTGAACGGGAAG Reverse: CGCCTGCTTCACCACCTTCT	[22]

A final volume of 20 μL was prepared in each tube by mixing 10 μL of SensiFAST™ SYBR^®^ Lo-ROX Master Mix Kit (Bioline Reagents Ltd, London, UK), 0.5 μL of each of the forward and reverse primer, 4 μL nuclease free water and 5 μL template or nuclease free water for non-template control (NTC) preparation. NTC samples were used both in PMA-treated and non-treated samples to confirm non - contamination of the PMA. The terms viability PCR and conventional PCR are used in this manuscript to refer to PCR with and without PMA treatment respectively.

A positive tissue culture was defined as the isolation of the same organism from two or more tissue samples. A cutoff value of 10^1^ cfu/mL for the sonicate culture positivity was used as recommended by Trampuz et al. [23]. Collectively, a positive culture per episode was defined as either two or more positive tissue samples and/or positive sonicate culture.

A positive PCR sample was defined as any significant amplification (using the dissociation curve and/or gel electrophoresis) with a Ct value above the detection threshold. Definition of a positive PCR episode is an episode with two or more positive tissue samples and/or positive sonicate in the same assay.

### Effect of PMA on sensitivity of PCR assay in clinical samples

Ten-fold serial dilution suspensions of fresh culture (18–24 h old) of *E. faecalis* were prepared from 10^8^ to 10^1^ cfu/mL. Non-infected tissue homogenates were tested with PCR to ensure they were *E. faecalis*-free. 500 mL of each bacterial suspension concentration was added to the same amount of tissue homogenate and then divided; one half was treated with PMA and the other left untreated. DNA was then extracted and PCR run as described above. Samples were run in duplicate in each experiment and three independent experiments were carried out on three different days. In addition to a positive control and NTC, a clean tissue sample was tested to further ensure lack of contamination. Ct values of PMA treated samples were compared to their untreated equivalents.

### Statistical analysis

Graphpad Prism 7 was used to analyse data and produce charts. Appropriate statistical tests were used according to the distribution of data. p values < 0.05 were considered significant. Using the above mentioned definitions, sensitivities, specificities, and accuracies of culture, PCR, PMA-PCR were calculated for the episodes rather than samples.

### Ethics

Human tissue samples were collected under the ethics approval of the Nottingham Health Science Biobank.

## Results

### PMA optimisation

**Fig. 2:**
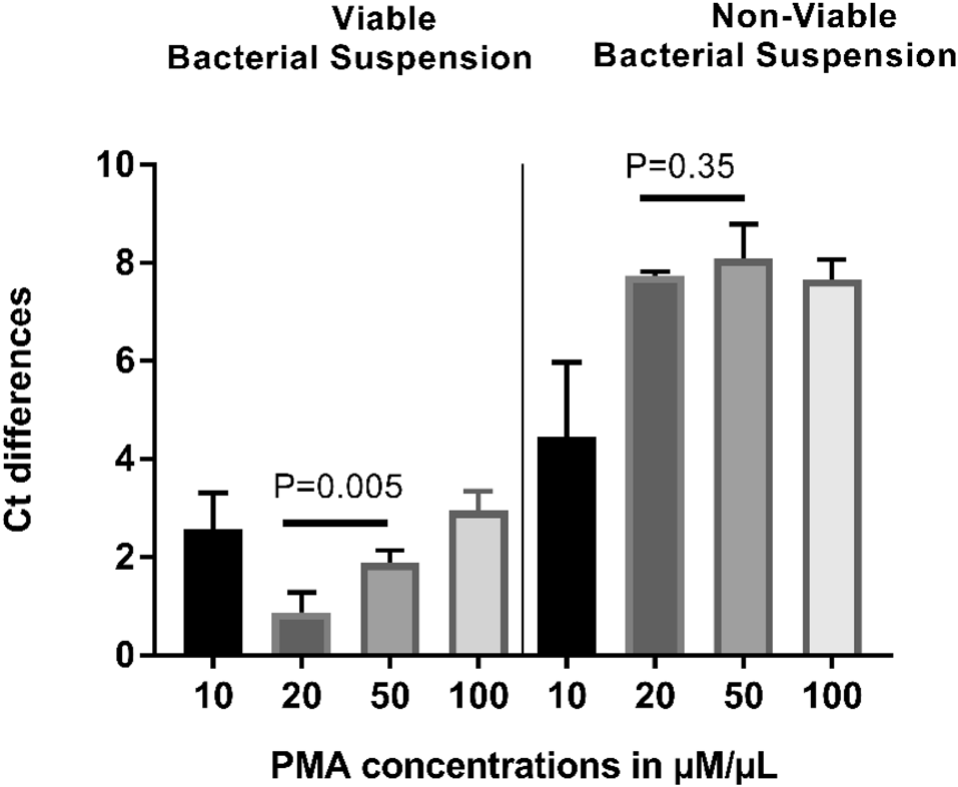
Optimisation of PMA: effect of PMA concentration

**Fig. 3:**
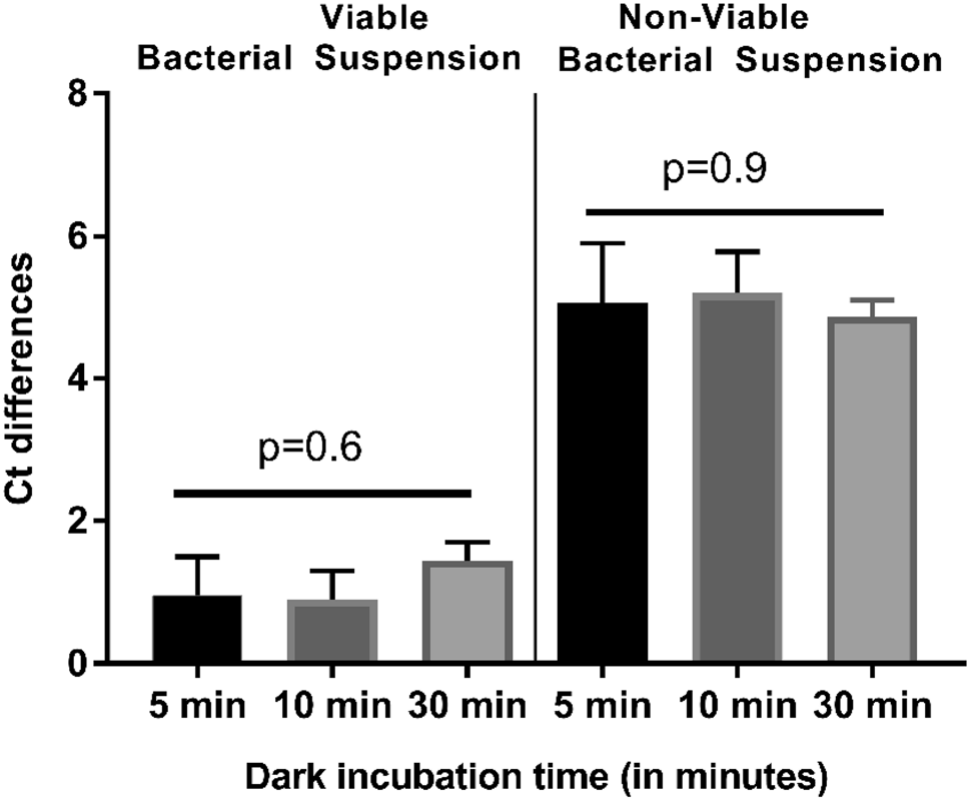
Optimisation of PMA: effect of dark incubation time

**Fig. 4:**
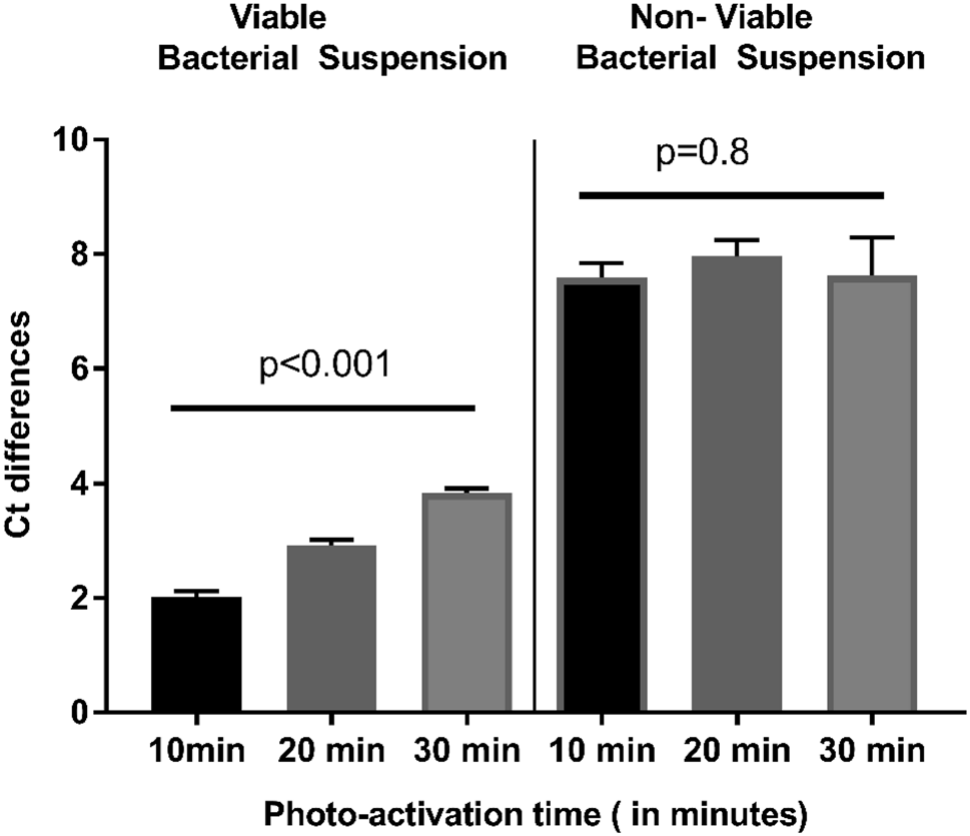
Optimisation of PMA: effect of photo-activation time

**Fig. 5:**
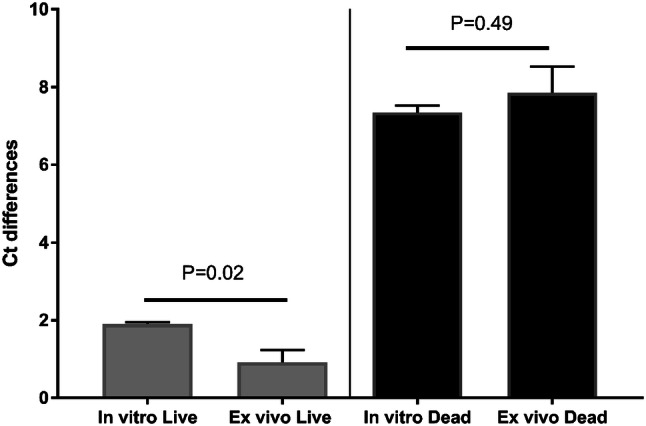
Optimisation of PMA: comparison of in vitro and ex vivo ΔCt viable and ΔCt dead in bacterial suspensions

**Fig. 6:**
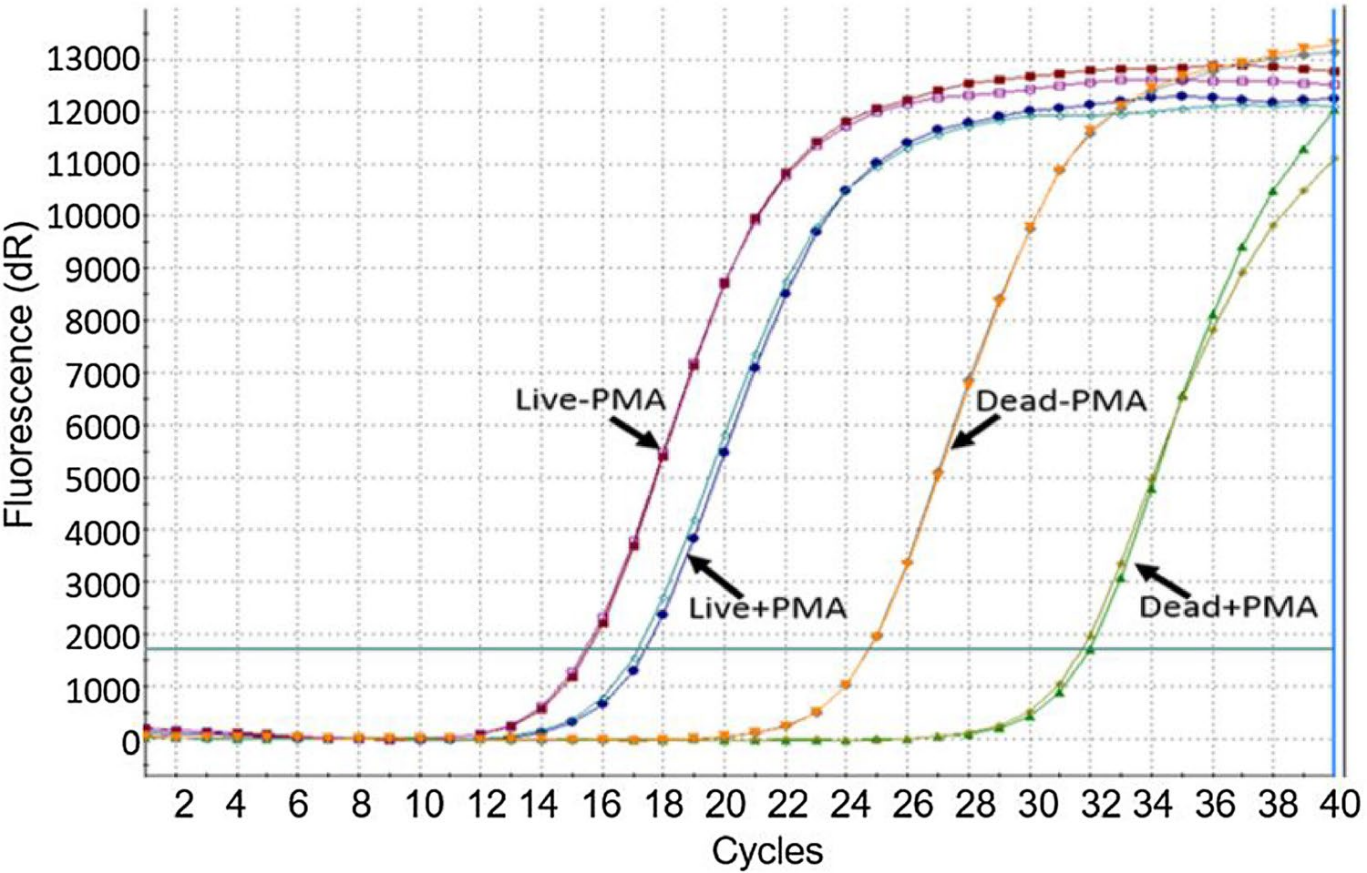
Optimisation of PMA: amplification plot of live and dead bacterial suspensions with/without PMA treatment

Figures 2, 3, 4, 5 and 6 show the results of PMA optimisation. ΔCt viable increased with the increase of PMA concentration from 20 to 100 μM. ΔCt dead increased with the increase in PMA concentration from 10 to 50 μM. The difference in ΔCt dead between 20 and 50 μM concentration was statistically non-significant (p=0.35). However, ΔCt viable varied significantly between these two concentrations. 20 μM was chosen to be used as it is the lowest concentration providing good live/dead discrimination (Fig. 1).

Differences in either ΔCt viable or ΔCt dead for different dark incubation times were not statistically significant (Fig. 2). The shortest tested time (5 min) was chosen for time-saving.

ΔCt viable, but not ΔCt dead, varied significantly between different photo-activation times (Fig. 3). As the ten-minute duration showed the lowest ΔCt viable, it was chosen to be the optimal photo-activation time.

### Application of optimised PMA in clinical samples

Fourteen arthroplasty revision episodes satisfied the MSIS definition of PJI. Nine of them had pus-draining sinuses at the time of presentation. In addition forty-six episodes of aseptic failure were recruited into this study. Demographics of participants are shown in Table 2.

**Table 2:** Demographics of participants

	PJI	Aseptic failures
Age in years		
Median	67.5	68
Range	51–83	42–91
Gender: no. (%)		
Male	7 (50)	19 (41)
Female	7 (50)	27 (59)
Arthroplasty site: no. (%)		
Hip	0 (0)	14 (30.4)
Knee	14 (100)	30 (65.2)
Ankle	0 (0)	2 (4.4)
Time since primary replacement in months^a^		
Median	26	120
Range	1–168	2–324
Number of revision: no. (%)		
First	5 (35.7)	36 (78)
Second	5 (35.7)	6 (13)
Third or more	4 (28.6)	4 (9)
Presence of a sinus: no. (%)		
Yes	9 (64.3)	0 (0)
No	5 (35.7)	46 (100)

^a^This data was unavailable in three episodes in the aseptic group

Sensitivity and specificity of culture methods was 50% and 98% respectively. *Staphylococcus epidermidis* and *E. faecalis* were isolated from three and two episodes respectively. *S. aureus*, *S. lugdunensis* were isolated from one episode each.

Of note, apart from the above mentioned 14 MSIS positive cases, there were two cases among the participants who were infected with streptococci (One with *Streptococcus agalactiae* and the other with *Streptococcus mutans*). These two cases were excluded from results analysis as these organisms are outside the panel of the PCR assays.

Among the 14 episodes of PJI included in the analysis, conventional PCR of periprosthetic tissues was positive in only two episodes while conventional PCR of sonicates was positive in nine episodes. Results of conventional and viability PCR are shown in Table 3.

**Table 3:** Results of conventional and viability PCR

Sample	Conventional PCR		Viability PCR	
	% Sensitivity (95% CI)	% Specificity (95% CI)	% Sensitivity (95% CI)	% Specificity (95% CI)
Periprosthetic tissue	15.38 (1.92 to 45.45)	100 (92.13 to 100)	23.08 (5.04 to 53.81)	100 (92.13 to 100)
Sonication fluid	64.29 (35.14 to 87.24)	56.52 (34.49 to 76.81)	71.43 (41.90 to 91.61)	82.61 (61.22 to 95.05)
Either of them	71.43 (41.90 to 91.61)	71.74 (56.54 to 84.01)	78.57 (49.20 to 95.34)	89.13 (76.43 to 96.38)

In this study, the use of PMA converted the positive PCR results of eight aseptic episodes into negative, thus, increasing the specificity of PCR from 71.7 to 89.1%. Interestingly, five out of these episodes had a suspicion of infection at some point of their clinical history (i.e. prolonged wound discharge or unexplained pain).

Ten samples (from eight episodes) tested positive with viability PCR and negative with conventional PCR. Seven and three out of these samples were in the enterococci and staphylococci assays respectively. Three of these episodes were revisions due to aseptic loosening. Other three episodes met the MSIS criteria and underwent first stage revision. One case was infected with positive intraoperative alpha-defensin testing but culture-negative. This case underwent debridement, antibiotics and implant retention (DAIR). The eighth episode was a re-implantation of presumably eradicated knee PJI case. This last case had polymicrobial infection and the sonicate tested positive for viability PCR of enterococci.

### Effect of PMA on sensitivity of PCR assay in clinical samples

Differences in Ct values between PMA-treated and untreated simulated infected human tissue samples for the concentrations between 10^8^ to 10^2^ cfu/mL were less than one cycle. However, the 10^1^ cfu/mL concentration was detected only in PMA-treated samples. The end reaction product was consistently higher for PMA-treated samples for the whole range of concentrations (p=0.01).

## Discussion

In the absence of a “gold standard” test, laboratory diagnosis of PJI continues to be challenging. The use of PCR has been investigated in an attempt to improve the diagnosis, but still has very limited clinical application. This study reveals a potential to overcome the indiscriminate detection of bacterial DNA in PCR by pre-treatment of samples with PMA.

PMA has been widely used to restrict the PCR detection to live cells, mainly in a laboratory setting. However, clinical as well as environmental samples pose a complex nature that could negatively affect the PMA performance, such as turbidity of the sample [12, 24] and organic material contents [25]. As PMA has the ability to react not only with DNA from different sources but also with various inorganic and organic molecules [16], clinical samples can have a deleterious effect on PMA, hence the importance of PMA optimisation for these samples. In addition, the matrix of biofilms contains bacterial extracellular DNA, and this has been found to bind to propidium iodide [26].

As shown in the PMA optimisation data, upon applying the same PMA conditions used in bacterial suspensions to simulated infected human tissue samples (i.e. ex vivo), the live/dead discrimination was not compromised. On the contrary, ΔCt viable was significantly lower. On further scrutinizing these results, amplification of the live bacterial samples was noted to be delayed when spiked in tissues compared to bacterial suspension, which could be explained by probable PCR inhibition by tissue products. This delay in amplification was less in samples treated with PMA than untreated samples which raises a possibility that PMA could be inhibiting the action of some PCR inhibitors. Although the mechanism is unclear the ability of PMA to react with various inorganic and organic materials might be an explanation. In the case of dead bacteria (with much less bacterial DNA available for amplification), there was an even earlier amplification for samples treated with PMA, a finding that strongly argued for desirable impact of PMA on the sensitivity PCR assay.

Unexpectedly, the sensitivity of the viability PCR was found to be higher than the conventional PCR (78.57% vs 71.43%). Ten samples tested positive with viability PCR and negative with conventional PCR. PMA might increase the sensitivity of PCR and lower its detection limit. PMA does not bind selectively to prokaryotic DNA and can interact with DNA from any source such as damaged eukaryotic cells [16]. PMA could therefore interact with the abundant human DNA in clinical samples preventing or limiting the generation and accumulation of PCR inhibitors, explaining the observed increased sensitivity. To investigate this hypothesis, an experiment was designed to study the effect of PMA on PCR in simulated clinical samples in an attempt to explain these unexpected results. Interestingly, the 10^1^ cfu/mL concentration was detected only by viability (PMA) PCR in our simulated infected clinical samples.

Our results of clinical samples showed that in periprosthetic tissues PCR has poor sensitivity (15.4% for conventional PCR and 23% for viability PCR) as previously found by Ryu et al. [27], Suda et al. [28], and Huang et al. [29]. They reported sensitivities of 16%, 30.8%, and 34% respectively. Several factors could contribute to the poor sensitivity of tissue PCR, including the small volume of samples used in PCR which makes it more liable to sampling errors. Difficult extraction of bacterial DNA because of the high viscosity of homogenates is another possible reason. Using larger volumes or higher concentrations may enhance the tissue PCR sensitivity. On the other hand, bacterial DNA in tissue samples would be overwhelmed with human genome DNA and loaded with PCR inhibitors. Potential abundance of PCR inhibitors in tissues also contributes significantly in this poor sensitivity. There is a need for techniques for purification of bacterial DNA and removal of human DNA for better results.

In this study, any amplification of the GAPDH gene was accepted as an indicator of lack of inhibition. We chose this external control (i.e. done in a separate aliquot) to avoid the potential interference of the target gene amplification by the co-amplification of the internal control [30].

With better understanding of the pathogenesis of PJI and biofilm development, retrieved prostheses have been appreciated as very valuable material for diagnosis. Sonication of prostheses has proved to be reliable, effective, and reproducible [23, 31]. Sonicates of implants have been used in both conventional and molecular microbiological methods, and more frequently than periprosthetic tissues as PCR material for PJI diagnosis, with superior sensitivity [27, 32, 33]. Our results agree with these studies as the sonicate PCR sensitivity was 64.3% and 71.4% for the conventional and viability PCR respectively compared to 15.4% and 23% for the periprosthetic tissues.

A major goal of this work was to determine the effect of prior PMA treatment of clinical samples on the performance of PCR in the diagnosis of PJI. Our results showed an increased specificity of PCR with PMA treatment.

Nocker et al. [15] has shown the usefulness of PMA in PCR studies in environmental situations, and Kobayashi [13] has shown its effect with PJI pathogens in vitro but recommended that it be validated with clinical samples. To the best of our knowledge, this is the first clinical study investigating the viability (PMA) PCR in the diagnosis of PJI. In this study, the use of PMA increased the specificity of PCR from 71.7 to 89.1% by converting the positive PCR results of eight aseptic episodes into negative. Interestingly, five out of these episodes had a suspicion of infection at some point of their clinical history (i.e. prolonged wound discharge or unexplained pain). In such cases, it is both challenging and crucial to decide how such a patient should be treated. Limiting the positive PCR results to viable bacteria could help to avoid unnecessary prolonged and costly treatment. On the other hand, persistence of the positive signal in PCR after PMA treatment, as in a further five aseptic episodes in our cohort, could identify these episodes as “at risk” and prompt closer and more frequent follow-up.

Our study has some limitations. Firstly, this is a single centre study with a relatively small number of participants. Small sample size resulted in wide confidence intervals of the calculated sensitivities and specificities. Secondly, the narrow PCR panel might have negatively affected the sensitivity. However, the primary focus of this research was to evaluate the effect of PMA on the performance of PCR in the diagnosis of PJI rather than testing the PCR per se. Furthermore, PMA optimisation was carried out for *S. aureus* only as it is one of the commonest causative pathogens of PJI. Different bacteria might require different optimised conditions. Other bacteria were not utilized in the optimisation process due to limited resources and they were found to work properly during the study. Multiple optimisations might deem necessary at a later stage.

In summary, pre-treatment of clinical samples with PMA has the potential to enhance both the specificity and sensitivity of PCR in the diagnosis of PJI. Further larger scale studies are needed prior to generalizing these finding and recommending the use of this technique in clinical settings.
