# Correction: Propidium monoazide–polymerase chain reaction for detection of residual periprosthetic joint infection in two-stage revision

**DOI:** 10.1007/s11033-024-09456-y

**Published:** 2024-04-20

**Authors:** Mohamed Askar, Mariam Sajid, Yassar Nassif, Waheed Ashraf, Brigitte Scammell, Roger Bayston

**Affiliations:** 1 Department of Academic Orthopaedics, Queen’s Medical Centre, University of Nottingham, C Floor, West Block, Derby Road, Nottingham, NG7 2UH UK; 2 Trauma and Orthopaedic Department, University of Nottingham Hospitals, Nottingham, UK

**Correction To: Molecular Biology Reports (2019) 46:6463–6470**

10.1007/s11033-019-05092-z

In this article the affiliation ‘Trauma and Orthopaedic Department, Faculty of Medicine, Mansoura University, Mansoura, Egypt’ for the author ‘Mohamed Askar’ was missing.

The original article has been corrected.

